# Predicting the Regulatory Dynamics of AML Disease Progression from Longitudinal Multi-Modal Clinical Data

**DOI:** 10.1007/s10916-025-02317-6

**Published:** 2025-12-13

**Authors:** Reza Mousavi, Moaath K. Mustafa Ali, Daniel Lobo

**Affiliations:** 1 Department of Biological Sciences, University of Maryland, Baltimore County, 1000 Hilltop Circle, Baltimore, MD 21250 USA; 2 Department of Hematology and Medical Oncology, Cleveland Clinic Taussig Cancer Institute, 9500 Euclid Avenue, Cleveland, OH 44195 USA; 3 Marlene and Stewart Greenebaum Comprehensive Cancer Center, Center for Stem Cell Biology & Regenerative Medicine, University of Maryland, School of Medicine, 22 S. Greene Street, Baltimore, MD 21201 USA

**Keywords:** Acute myeloid leukemia, Disease progression prediction, Systems medicine, Mechanistic clinical modeling, High performance computing, Longitudinal Multi-Modal data

## Abstract

Acute Myeloid Leukemia (AML) is a complex and heterogeneous disease identified by severe clinical progression, fast cellular proliferation, and often high mortality rates. Incorporating diverse longitudinal information on patients’ medical histories is essential for developing effective disease predictive models applicable to both research and clinical settings. Here, we present a robust methodology for discovering the regulation of disease progression dynamics from a novel longitudinal, multimodal clinical dataset of patients diagnosed with AML. The medical data were analyzed to reveal the main clinical, genetic, and treatment features modulating disease progression. To discover dynamic mathematical models at the systems level—including the necessary regulatory interactions, parameters, and disease drivers—predictive of AML progression, we developed a *de novo* inference algorithm based on high-performance evolutionary computation. The results demonstrate that the predictive methodology could accurately estimate the drivers and clinical dynamics of AML progression in terms of blast percentages for both training and novel patients. This approach effectively predicted AML drivers, their mechanistic interactions, and disease progression by leveraging the heterogeneous and longitudinal dynamics of patients’ clinical data. Importantly, this methodology shows significant potential for modeling progression dynamics in other acute diseases, providing a flexible and adaptable framework for advancing clinical and translational research.

**Supplementary Information:**

The online version contains supplementary material available at 10.1007/s10916-025-02317-6.

## Introduction

Acute myeloid leukemia (AML) is an aggressive and heterogeneous malignancy of the myeloid lineage, characterized by clonal expansion of abnormal precursor cells and impairment of normal hematopoiesis [1]. AML is driven by different genetic and cytogenetic abnormalities within myeloid precursors that result in neoplastic transformation [2–4]. Poorly differentiated myeloid cells can accumulate in the bone marrow, their site of origin, and circulate in the peripheral blood. This can cause multiple complications such as increased risk of infections, bleeding, fatigue, and bone pain [5, 6]. The incidence of AML has continued to increase over the past few decades [7]. The American Cancer Society estimates approximately 59,610 new cases and 23,710 deaths due to leukemia (all types) in the United States in 2023. Among these, approximately 20,380 new cases are expected to be diagnosed with AML, mostly in adults, resulting in 11,310 deaths. AML accounts for approximately 1% of all cancers and is more common in adults over the age of 45 years; however, AML can also occur in children. AML progresses rapidly, with a 5-year survival rate of approximately 28% in adults [8]. Hence, the rapid and precise prediction of disease progression is essential for cost-effective treatment decisions and improving patient outcomes.

Owing to its significant contribution to predictive medicine and modern oncology, machine learning (ML) has recently gained increasing attention in cancer research [9–14]. Recent studies have demonstrated that ML has excellent capabilities for handling large amounts of complex data and may prove to be a powerful tool for understanding and treating diseases [15–18]. In AML, a highly heterogeneous hematologic malignancy, interpreting diagnostic tests, such as genetic mutations, chromosomal abnormalities, and blast percentages, has historically required experienced clinicians with years of training and expertise. However, the landscape of AML diagnosis and management has significantly changed with the advent of ML algorithms [19–21]. Recent ML algorithms have been applied to several tasks in AML, including the initial diagnosis [22–24], marker and therapeutic target discovery [25, 26], and prognosis and treatment response estimation [27–31]. However, existing ML models lack explainability, making it difficult for clinicians to understand how specific markers or interactions contribute to predicting disease prognosis. This limits the applicability of such models in clinical practice, where interpretability is critical for decision-making. Furthermore, these ML approaches rely on static data and lack the capability to incorporate temporal information from patient medical records to predict disease progression. Such static models overlook the dynamic nature of AML, in which disease progression and treatment response evolve over time.

Dynamic mathematical models have been proposed to predict AML disease progression from clinical data [32, 33]. These approaches can simulate the dynamics of leukemic cells during the clinical history of patients and predict disease prognosis [34, 35], treatment scheduling [36, 37], transplant dosage [38], and specific therapy response [39]. However, several significant challenges remain. One major challenge lies in the complexity of managing and integrating high-dimensional, multimodal longitudinal clinical data into a mathematical dynamic model, which includes patient information, leukemia-associated genetic factors, and treatment responses collected at different time points. Conventional temporal modeling methods struggle to effectively integrate and analyze these large and diverse data types, often leading to suboptimal results with limited clinical relevance. To address these gaps, innovative methodologies are needed that can efficiently process and analyze multimodal data on a large scale while providing mechanistic insights with dynamic models of AML progression.

Here, we propose a *de novo* inference methodology to derive systems-level dynamic models of AML progression from longitudinal, multimodal data. This innovative approach integrates the strengths of evolutionary computation, high-performance computing, and mathematical modeling [40–44] to predict disease progression dynamics. The proposed methodology infers models that include both the topology and parameters of a dynamic system, represented by a system of ordinary differential equations (ODEs). By simulating these ODE-based models, the approach effectively recapitulates and predicts observed disease progression dynamics from clinical data, thereby providing a framework for understanding and predicting AML trajectories. To evaluate the approach, dynamic models were inferred from a newly curated AML clinical dataset collected at the University of Maryland Medical Center. These findings demonstrate the effectiveness of the methodology in inferring disease drivers and mechanistic interactions from longitudinal clinical data and in accurately predicting dynamic disease progression in new patients. This study establishes a foundation for advancing AML prognosis, guiding personalized treatment strategies, and improving patient outcomes, with potential applicability in modeling progression dynamics of other acute diseases.

## Methods

### Mechanistic Inference of AML Progression Dynamics from Multimodal Clinical Data

**Fig. 1:**
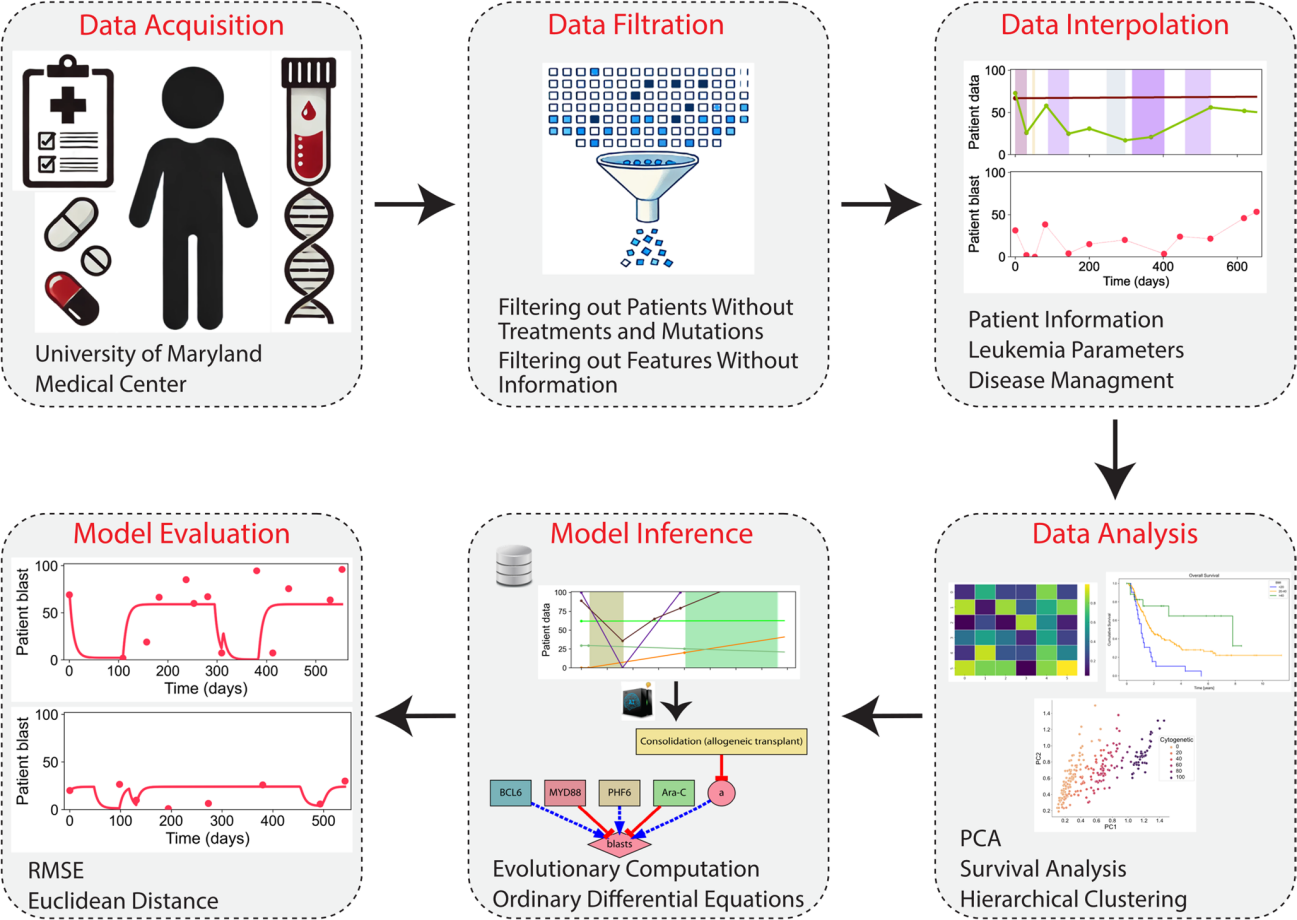
Methodology for the automatic inference of mechanistic, dynamic AML progression models. The method consists of six stages: data acquisition (collecting clinical data, including patient information, genetic abnormalities, and treatment information), data filtration (removing patients and features with missing or insufficient information), data interpolation (generating continuous temporal representations of multimodal clinical features), data analysis (applying techniques such as Principal Component Analysis (PCA), survival analysis, and clustering), model inference (discovering dynamic models with evolutionary computation and systems of differential equations), and model evaluation (assessing model performance with metrics such as Root Mean Square Error (RMSE) and Euclidean distance)

Leveraging large-scale multimodal clinical data alongside longitudinal aspects of patients’ medical records is essential for advancing personalized medicine to address unmet clinical demands in acute diseases. This approach facilitates a deeper understanding of disease progression and paves the way for the development of tailored interventions to improve patient outcomes. To this end, we developed a computational pipeline for the analysis and mechanistic *de novo* inference of AML progression dynamics from multimodal clinical data, including patient information, leukemia parameters, and disease management strategies. The proposed pipeline comprises several key steps: data curation, data filtration, data interpolation, data analysis, model inference, and model evaluation (Fig. 1). The inferred interpretable models consist of a system of dynamic mathematical equations including quantitative input nodes, which include patient information, leukemia parameters, and treatment interventions, together with inferred intermediate nodes. The output node predicts the temporal dynamics of disease progression, measured in AML as the percentage of blasts abnormally present in the bone marrow or blood. The models also include mechanistic regulatory interactions, allowing nodes to positively or negatively affect other nodes. The intermediate and output nodes are capable of self-regulation and can also regulate other nodes. However, the input nodes cannot be affected by regulations from other nodes. This design allows the systems-level models to capture the complex regulatory dynamics underlying disease progression. A *de novo* inference method was developed using high-performance evolutionary computation [42] to efficiently discover dynamic models (see Supplementary Information for details). These models include the number of nodes, their regulatory interactions, and parameters derived from the clinical dataset. The method can infer the topology and parameters of a dynamic predictive model defined by a system of ordinary differential equations (ODEs), which can accurately simulate AML disease progression, defined as the blast percentage over time. Taking the curated longitudinal multimodal dataset as input, the method can provide a reliable and interpretable framework for understanding and predicting individual disease trajectories.

### Data Collection and Curation from an AML Patient Cohort

Anonymized data from patients diagnosed with AML between October 2006 and June 2021were collected from the University of Maryland Medical Center, comprising 467 patients and 125 multimodal variables, including patient, molecular, cytogenetic, and treatment data. Patient information was collected from the date of diagnosis to the date of death or last follow-up. All therapies administered and corresponding responses, assessed using ELN 2017 criteria, were collected [45]. Moreover, blast percentage, cytogenetic exam, and molecular testing using next-generation sequencing (NGS) or PCR-based fragment analysis were collected at each response examination when available. Blast percentages were determined based on bone marrow (BM) aspirate and biopsy data or/and peripheral blood (PB). All data were stored in REDCap and were updated until March 2022 [46]. Notably, each type of treatment was considered as a separate feature, but patients could receive combinations of treatments simultaneously or at different times throughout their clinical history. To ensure the integrity and quality of the analysis, a rigorous data filtration process was implemented. 19 patients were excluded due to missing treatment information and 18 patients were excluded due to inconsistencies in the treatment dates. Additionally, 80 patients were excluded due to missing mutation information, and 145 patients were filtered out due to insufficient or missing data in a disease progression marker—blast percentage. Predictive features lacking informative value were then removed, resulting in the exclusion of 50 features from the dataset. After filtration, the final curated clinical dataset comprised 245 patients with 75 predictive features, resulting in a robust and high-quality cohort for further analysis. A summary of the predictive features is presented in Table 1 (see Supplementary Information for additional details and Supplementary Fig. 1 for a patient flow diagram illustrating the cohort selection process).

**Table 1:** Features in the curated multimodal AML clinical dataset used for analysis and dynamic model training

Category	Number of Features	Features	
Patient Information	3	Age, ECOG, BMI (Body Mass Index)	
Leukemia Parameters	60	Cytogenetic Abnormalities, Genetic Mutations (FLT3-ITD, FLT3-TKD, ASXL1, ASXL2, BCOR, BCORL1, BRAF, BRINP3, CALR, CBL, CEBPA, CSF3R, DDX41, DNMT1, DNMT3A, EED, ELANE, ETNK1, ETV6, EZH2, FLT3, GATA2, HNRNPK, HRAS, IDH1, IDH2, IKZF1, JAK2, JAK3, KIT, KMT2A, KRAS, MPL, NOTCH1, NPM1, NRAS, NSD1, PHF6, PRPF40B, PRPF8, PTPN11, RAD21, RUNX1, SETBP1, SF1, SF3A1, SF3B1, SH2B3, SMC1A, SMC3, SRSF2, STAG2, SUZ12, TET2, TP53, U2AF1, U2AF2, WT1, ZRSR2)	
Disease management	12	Conventional Chemotherapy	Anthracycline-based, Ara-C, CLAG-M/FLAG-IDA, Cladribine-based
		Drug Therapy	Hypomethylating agent (HMA), Asparaginase-based, FLT3 inhibitors (FLT3i), Venetoclax-based (VEN), IDH1/IDH2 inhibitors (IDH1/IDH2i)
		Cellular therapies	Consolidation (allogeneic transplant), Donor lymphocyte infusion (DLI)
		Others	

**Fig. 2:**
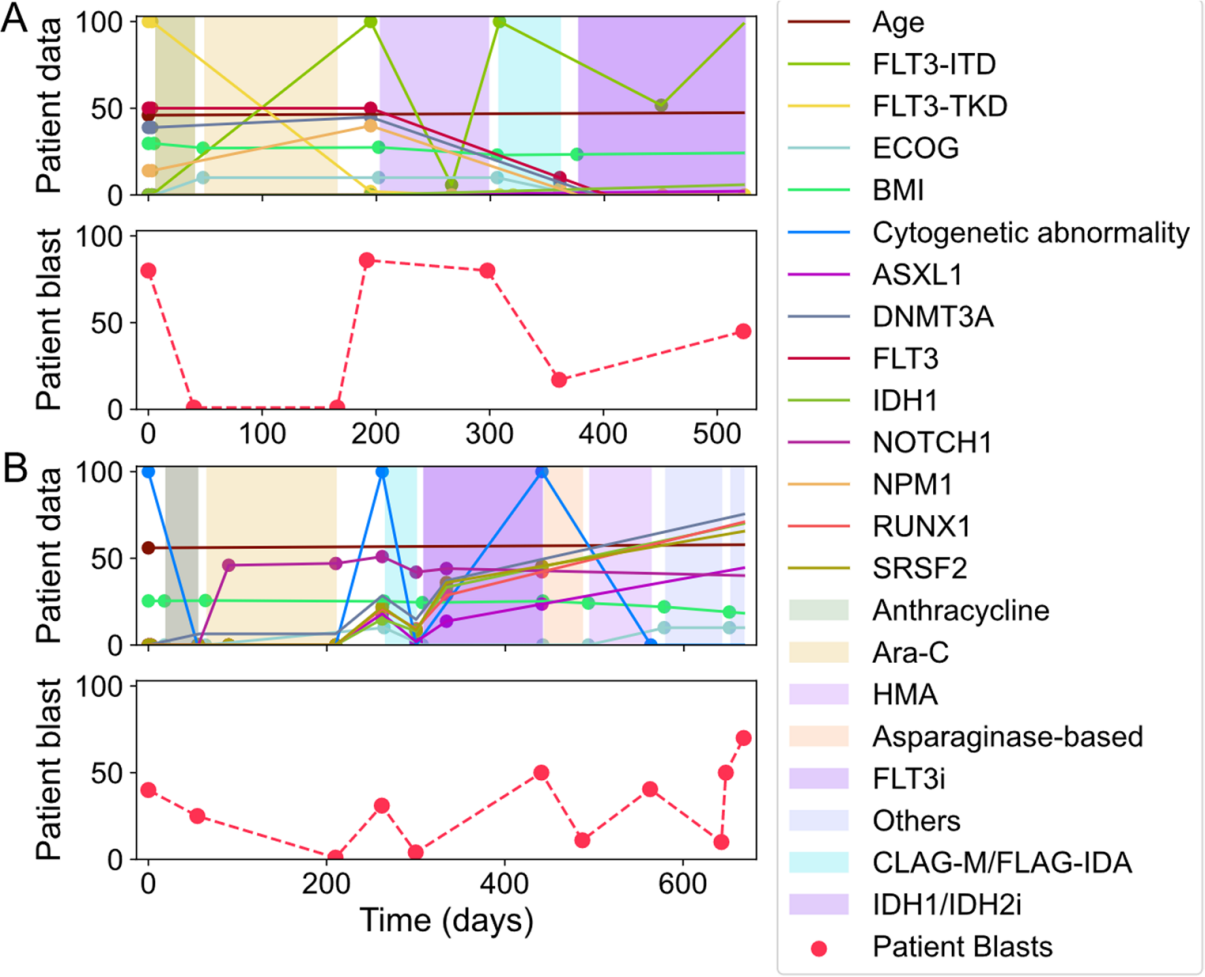
Longitudinal dynamics of cancer progression for two patients (**A** and **B**). Top panels for each patient illustrate longitudinal changes across three input modalities: (i) Demographic and clinical features, including age, BMI, and ECOG performance status; (ii) leukemia-associated genetic features, such as cytogenetic abnormality and mutation status of genes including FLT3, NPM1, IDH1, TP53; (iii) treatment interventions, shown as shaded regions corresponding to the treatment periods for each patient. Bottom panels for each patient represent the longitudinal dynamics of disease progression, measured by the percentage of blasts over time

Figure 2 illustrates the dynamics of leukemia progression in two patients after integrating and interpolating patient information, leukemia-associated genetic parameters, and treatment interventions over time. The top panels for each patient depict longitudinal changes in clinical parameters (e.g., age, Body Mass Index (BMI), ECOG performance status), genetic mutations (e.g., FLT3-ITD, DNMT3A), and cytogenetic abnormalities, along with the timing and type of treatments. Treatment periods are represented by shaded regions corresponding to specific therapeutic windows. The bottom panels show disease progression as the percentage of blasts, a key marker of leukemia. Patient A experienced an initial decline in blast percentage with Anthracycline and Ara-C (chemotherapy drugs commonly used to treat AML) treatments, followed by a relapse. Patient B showed more fluctuation in both cytogenetic abnormalities and genetic mutations, with alternating periods of remission and progression aligned with treatment periods. These results highlight the complex interplay among individual patient factors, genetic and chromosomal abnormalities, and therapeutic responses in the prognosis and management of AML.

## Results

### Analysis of the Curated Clinical Dataset

**Fig. 3:**
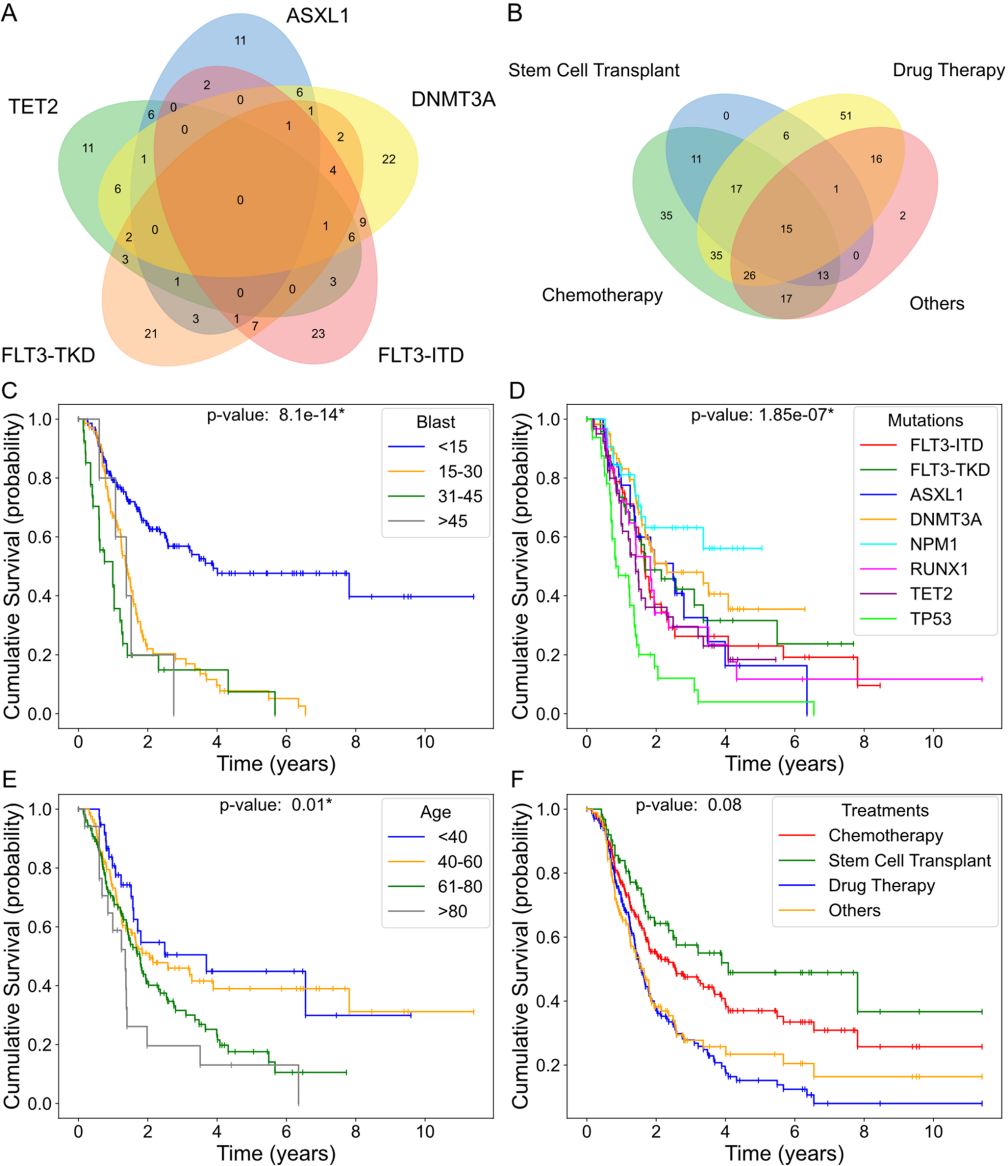
Venn diagrams and survival analysis of patient cohorts based on clinical, molecular, and disease progression features. (**A**) Venn diagram illustrating the overlap of key genetic mutations in AML. (**B**) Venn diagram showing the overlap among different treatment approaches. **C-F.** Kaplan-Meier survival curves illustrating cumulative survival stratified by blast percentages (**C**), gene mutations (**D**), age groups (**E**), and treatment regimens (**F**). The Venn diagrams as well as the treatment and mutation survival curves were generated using binary data. The age survival curves were stratified by patients’ age at the time of diagnosis. The blast curves were derived from continuous data, calculated as the area under the curve (AUC) divided by the observation duration (in days). Statistical significance among the survival groups was assessed using log-rank tests, with significance defined as $\:p\:<\:0.05$ and indicated by *

We analyzed key features curated in the clinical dataset to illustrate the overlap between critical mutations and treatment approaches in patients with AML. Figure 3 A highlights the overlap among frequently observed mutations across the patient cohort. Each overlapping region indicates the number of patients with specific combinations of mutations during their clinical history. Interestingly, six patients developed mutations in three genes concurrently: *DNMT3A*, *FLT3*-ITD, and *TET2*. Figure 3B shows the overlap among the four general classifications of the 14 treatment approaches included in the dataset. Each intersection in the Venn diagram shows the number of patients who received each combination of different treatment types during their AML clinical history. Notably, 15 patients received all four treatment strategies.

Next, we analyzed the curated dataset to reveal cumulative survival curves of patients with different clinical features. Analysis of survival curves by blast counts revealed that patients with less than 15% blast counts had the highest survival probability. However, all groups with blast counts higher than 15% had significantly lower survivability, independent of their counts (Fig. 3C). Analysis of the survival probabilities by the main genetic mutations demonstrated a significant effect of different mutations on patient survival, where mutations in *TP53* (*p53*) resulted in the lowest survival probability (Fig. 3D). Figure 3E shows the survival curves by age, highlighting the significant decline in survivability of patients older than 61 years. Finally, Fig. 3F compares survival rates across different treatment types. Although their differences were not significant, stem cell transplantation resulted in the highest survival rates, followed by chemotherapy, drug therapy, and others.

**Fig. 4:**
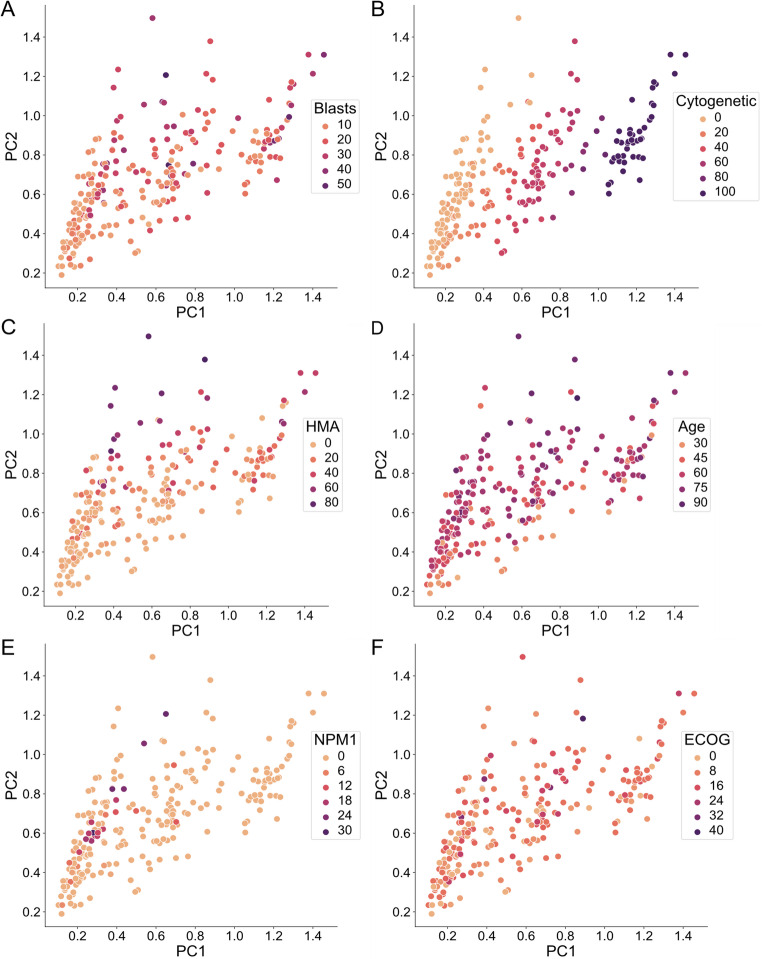
PCA plots exploring the relationships between clinical and molecular features and disease progression in AML.(**A**) Blast percentage, representing a marker of disease progression. (**B**) Cytogenetic abnormalities, reflecting changes in chromosome number or structure. **(C)** Hypomethylating Agents (HMA), a class of drugs used in AML treatment. (**D**) Age at time of diagnosis. (**E**) Mutations in nucleophosmin 1 (*NPM1*), (**F**) ECOG performance status, a functional measure of daily activity and self-care ability. For each feature, except age, data were derived from dynamic variables and calculated as the area under the curve (AUC) divided by the clinical history duration. Each point represents an individual patient, plotted according to their distribution across the first two principal components (PC1 and PC2). Points are colored by the respective clinical or disease feature to highlight potential correlations

To examine possible correlations between clinical features and disease progression (blast percentage), a principal component analysis (PCA) was performed on the curated AML clinical dataset. Figure 4 shows the PCA plots, highlighting the distribution of selected features across patients. From this analysis, we observed that patients with worse disease progression (i.e., high blast percentage) did not cluster separately from those with better prognosis (Fig. 4A). Instead, the main driver of the first principal component (PC1) was cytogenetic abnormality level (Fig. 4B). However, it did not correlate with the blast percentage (Fig. 4A). The main driver of the second principal component (PC2) was hypomethylating agents (HMA) (Fig. 4C), which did not correlate with the disease outcomes (Fig. 4A). The results showed that the remaining features did not cluster or correlate with disease outcomes (Fig. 4D-F). Additional analyses with non-linear dimensionality reduction (UMAP) resulted in similar non-clustered patterns for all features. Although cytogenetic abnormalities and HMA showed slight separation, they still did not correlate with blast percentages (Supplementary Fig. 2). Similarly, a clustered heatmap including all features in the clinical dataset revealed no strong correlations between the different features or blast percentages (Supplementary Fig. 3). Together, these results suggest that no strong clustering or correlation exists between clinical features and disease progression when analyzed using linear and nonlinear methods.

Further, two multivariable approaches based on supervised machine learning (ML), Random Forest and Gradient Boosting ensemble algorithms [40], were tested to evaluate the predictability of the curated AML dataset. The normalized data were split into 90% for training and 10% for testing. The resulting RMSE on the testing set was 15.23% for Random Forest and 15.01% for Gradient Boosting. Crucially, despite showing moderate performance in predicting blast percentages, these ML models were trained on static data, where each sample (patient) was represented by a single average value for each feature (Table 1). As such, they do not incorporate temporal information from patient medical histories, which is essential for modeling disease progression. Collectively, the findings from PCA, UMAP, and ML analyses highlight the need for advanced computational approaches based on dynamic mathematical models to discover predictive mechanisms of AML disease progression from clinical data.

### A Novel *de novo* Inference Algorithm for Dynamic Mechanistic Models of Disease Progression

We developed a *de novo* inference algorithm to automatically discover dynamic mechanistic models of AML progression from patient clinical outcomes. The predictive models are formulated as a system of nonlinear ordinary differential equations (ODEs) to capture the temporal dynamics of disease progression. Each model includes three node types. Input nodes incorporate patient information, leukemia-associated genetic parameters, and treatments derived from the clinical data. To enable temporal modeling, discrete clinical data points were transformed into continuous temporal trajectories using linear interpolation, allowing the model to track changes in feature values throughout each patient’s clinical history. Nodes in the middle layers process and integrate these input signals. The output node predicts how the disease progresses over time, as quantified by the percentage of abnormal cells (blasts). Links between nodes indicate nonlinear regulatory interactions from different features to the intermediate and output nodes. Such interactions include different numeric parameters specifying their regulatory strength and can be positive or negative and grouped in a necessary or sufficient fashion. Taking the interpolated clinical history of a patient as input, an ODE model, when simulated, can precisely and quantitatively predict the temporal progression of the disease (as measured by blast percentage).

To infer *de novo* an optimal interpretable model, including the necessary disease drivers and their mechanistic interactions, we applied high-performance evolutionary computation to clinical datasets. The algorithm starts with a random population of predictive models. Each model comprises input signals derived from the clinical data, an output node predicting blast percentage, and a random number of intermediate nodes. In addition, random nonlinear regulatory interactions (excluding regulators for input signals) and random parameters are assigned to each initial model. These initial models are then translated into a system of ordinary differential equations and numerically simulated with the clinical data of each patient included in the training set. Their predictive accuracy is assessed using an error function that quantifies the difference between predicted and observed disease progression. Models that better recapitulate the observed disease progression (blast percentage over time) for the patients are preserved in the population, while those with worse errors are eliminated. The preserved models produce new offspring models through stochastic crossover (combining two parent models) and random mutations (changing parameters and adding or deleting links or nodes). These offspring models are subsequently simulated, scored, and integrated into the population for the next selection cycle. This iterative process continues until a model with zero error is identified, representing an accurate depiction of the temporal dynamics of disease progression. Notably, during evolution, the method automatically selects only the clinical features necessary to recapitulate disease progression, resulting in a final model that includes only driver inputs.

### Inferred Dynamic Mechanistic Models of AML Progression from Clinical Data

**Fig. 5:**
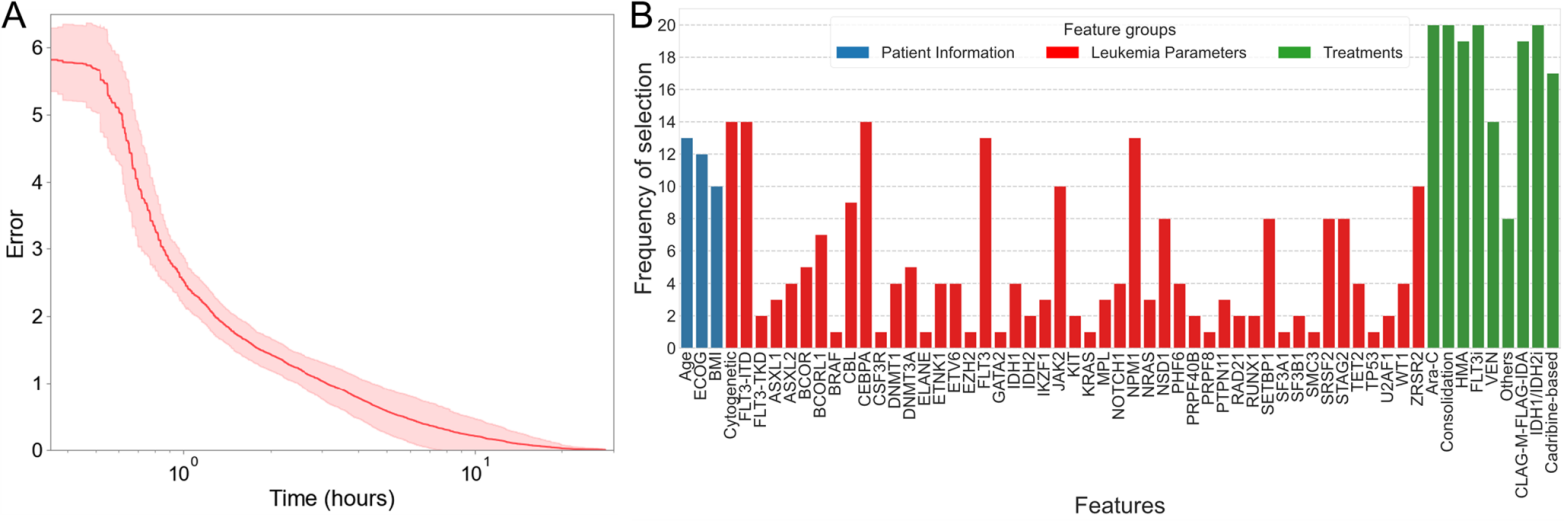
Overview of evolutionary dynamics and feature selection frequency over 20 independent runs of the inference methodology. (**A**) Error trajectory of the best-performing model on the training dataset. The fitness error decreases consistently, converging to zero after approximately 24 h of execution. The line and shaded area indicate the average and standard deviation, respectively, of the error values across all runs. (**B**) Frequency of feature selection across the 20 independent training runs. Feature selection frequency reflects how often a feature was retained in the final model across repeated runs, serving as a proxy for predictive stability and importance. Features are grouped into three categories: (i) Patient information (blue), including age, BMI, and ECOG performance status; (ii) leukemia-specific molecular parameters (red), such as recurrent genetic mutations and cytogenetic abnormalities; (iii) treatment exposures (green), including agents such as Ara-C and HMA. For each run, training data were generated by randomly shuffling the dataset and selecting 90% of the samples.

To infer mechanistic models of AML progression, we executed the proposed algorithm in 20 independent runs. In each run, 90% of patients from the curated clinical dataset were randomly selected for training, while the remaining 10% were held out for testing. Figure 5A shows the overall evolutionary dynamics of the algorithm across the 20 independent runs. The results indicated that after approximately 24 h of computation, all the evolutionary processes converged, yielding models capable of recapitulating disease progression dynamics with zero fitness error. The performance of the models resulting from each run was evaluated using five common metrics: root-mean-square error (RMSE), normalized root-mean-square error ($\:{RMSE}_{n}$), coefficient of determination ($\:{R}^{2}$), mean absolute percentage error (MAPE), and mean absolute error (MAE). These metrics were selected to capture both absolute and relative prediction accuracy, as well as the overall goodness-of-fit across longitudinal prediction tasks. Specifically, MAPE and $\:{RMSE}_{n}$ allow for scale-invariant evaluation, while R² provides an interpretable measure of the proportion of variance explained by the model. On average, the training RMSE was 9.48%, and the testing RMSE was 11.62%. The $\:{RMSE}_{n}$ was 1.15% for training and 1.42% for testing. The R² values were 0.78 for training and 0.72 for testing, indicating good overall performance and generalizability. The MAPE was 1.55% for training and 1.87% for testing. The MAE was 6.37% and 7.62% for the training and testing sets, respectively. These results highlight the strong generalization capability of the method, as evidenced by the relatively minimal differences between training and testing performance. Moreover, the proposed method outperformed both random forest and gradient boosting methods in terms of RMSE. The average model complexity (number of nodes and links) was 57, achieving a balance between capturing the dynamic dataset and maintaining interpretability.

Figure 5B illustrates the frequency of feature selection across the 20 runs of the proposed methodology, identifying the predictive features that significantly contribute to the dynamics of AML disease progression. Notably, chemotherapy (Ara-C, other than high dose-Ara-C), consolidation therapy (high dose Ara-C), FLT3 inhibitors (FLT3i), and IDH inhibitors (IDH1/IDH2) were selected to be included in the inferred model for every run, emphasizing their critical importance for AML management. Additionally, the predicted features included other key factors, such as age, ECOG performance status, and cytogenetic abnormalities, as well as *FLT3*-ITD, *CEBPA*, and *NPM1* mutations, as primary drivers of AML progression. Overall, this analysis revealed the most relevant features influencing AML progression, as identified by the inferred predictive models

### Mechanistic Insights into AML Progression and Patient-Specific Dynamics

**Fig. 6:**
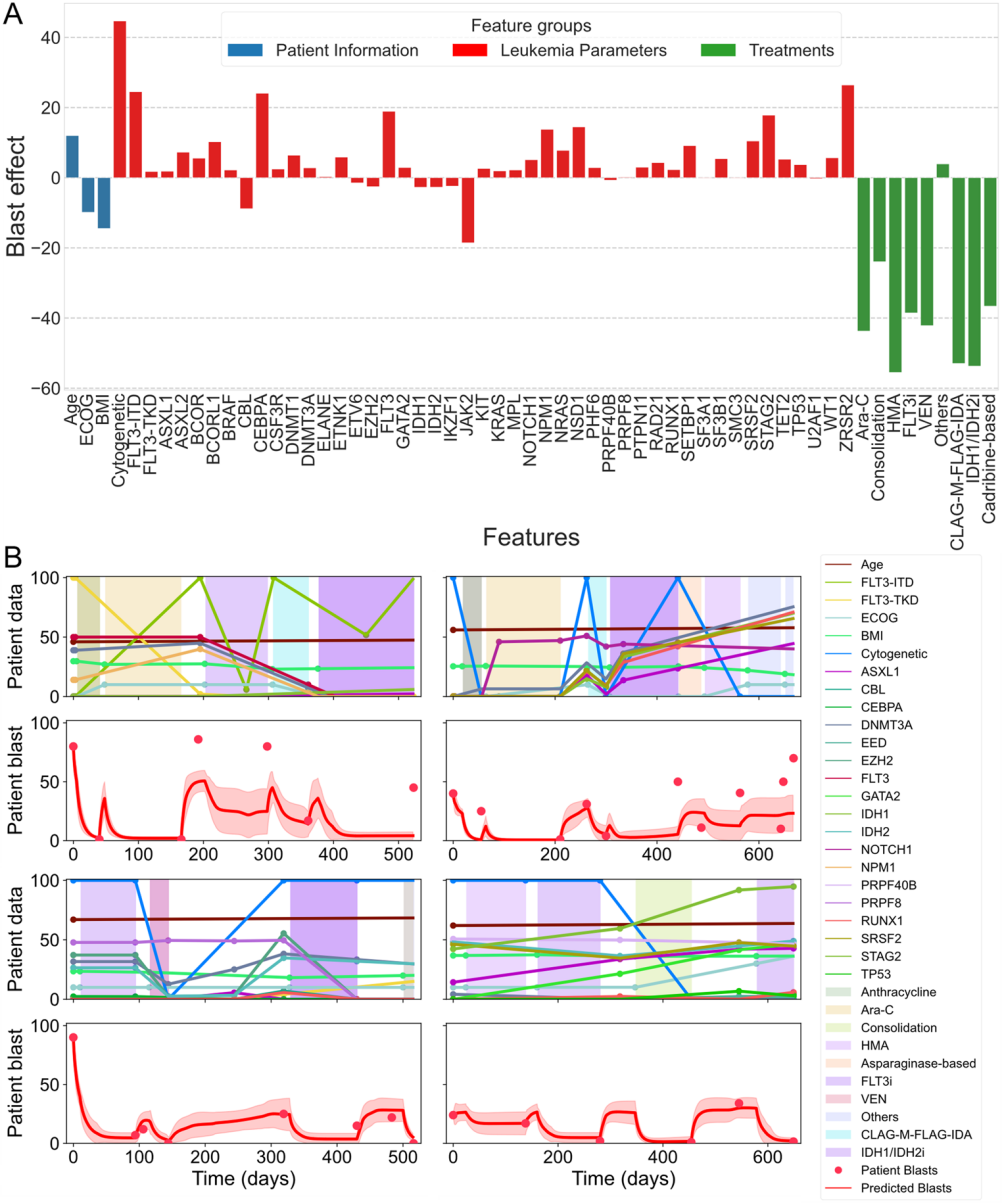
Overview of clinical feature effects on blast percentages and predicted patient blast dynamics from the inferred mathematical models. (**A**) Predicted mechanistic regulation effects of clinical features on blast percentages. (**B**) Predicted disease progression dynamics for four patients in the curated dataset. The line and shaded region represent the average and standard deviation, respectively, of error values across all models

Analysis of the inferred regulatory effect of the selected features across all runs on blast percentages revealed the predicted drivers of AML disease progression. Figure 6A illustrates the mechanistic regulation strength of each feature over the 20 independent runs of the predictive methodology. Each bar represents a feature’s overall impact on the blast percentages, computed as the normalized link strength. This value is computed as the average of the half-response parameters and sign as the multiplicative positive or negative effect in a pathway across all predictive models. The results showed that nearly all treatments together with ECOG performance were identified by the predictive models as having a strong negative effect on blast percentages, indicating a positive correlation with improved AML patient outcomes. Conversely, cytogenetic abnormalities, as well as mutations in *FLT3*-ITD, *CEBPA*, *NPM1*, *ZRSR2*, and *FLT3*, were predicted to have a strong positive effect on blast percentages, suggesting their roles as major drivers of AML progression. Overall, this analysis highlighted the predicted effects of link strength from predictive features on the blast percentages, as identified by the models.

Figure 6B illustrates the overall disease dynamics of the models, averaged across the 20 independent runs, for four individual patients from the curated dataset (see Supplementary Fig. 4 for all patients). The patient-specific blast dynamics highlight both the consistency and variability of predictions across different runs over time. The observed trends demonstrate how all models effectively captured the dynamics of individual patient blast percentages while adapting to the variability introduced by training data randomization and stochastic model initialization. These findings provide valuable insights into disease progression, laying the groundwork for the development of tailored patient-specific predictive strategies.

**Fig. 7:**
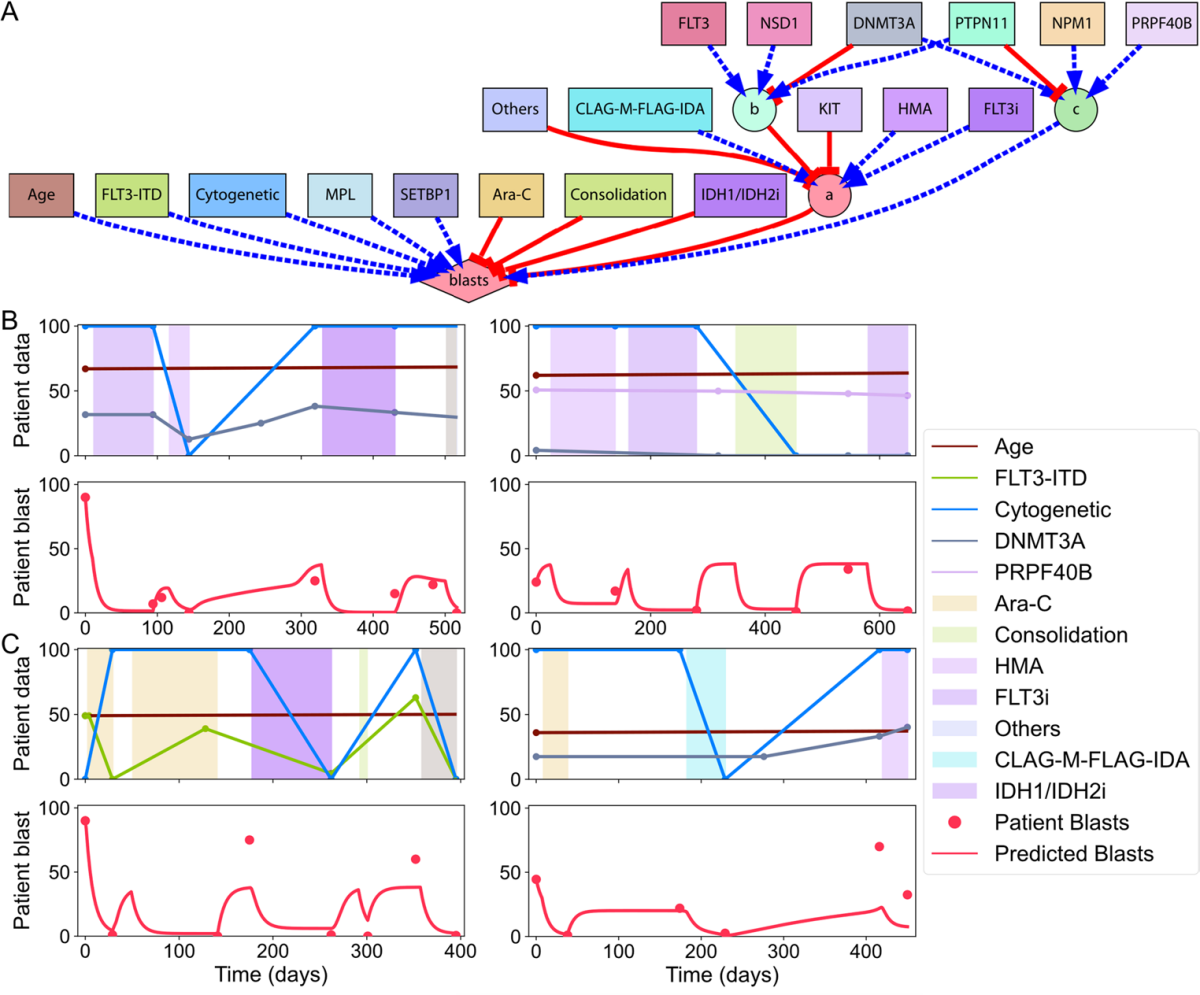
Inferred model dynamics for AML disease progression.** A**. The dynamic model inferred by the predictive methodology incorporates input features (squares), intermediate variables (circles), and an output (diamond) node. Regulatory interactions are represented as either positive (pointed arrows) or negative (blunt arrows) and grouped as necessary (solid lines) or sufficient (dashed lines). **B-C**. Simulations of the best model. (**B**) shows two patients from the training dataset, highlighting the predicted blast percentages over time, while (**C**) depicts two patients from the test dataset, highlighting model performance on unseen data. Curve, line, and shaded area colors in (**B**) and (**C**) correspond to the node colors in the model network diagram in (**A**)

Figure 7 illustrates the best-performing dynamic model, including its mechanistic regulations and simulations for two different patients (see Supplementary Information for the model system of equations). As shown in Fig. 7A, the discovered model has a total of 47 components (nodes and regulatory links) and includes three intermediate nodes to integrate the different mutation and treatment effects into the blast dynamics. Figure 7B-C demonstrates the model simulations for two patients from the training and test datasets, respectively, highlighting its accuracy in capturing both observed and unseen patient dynamics. These results indicate that the proposed predictive methodology can not only accurately model the progression of AML but also generalize well to new patient data, demonstrating its ability to understand and forecast disease dynamics in clinical applications.

## Discussion

In this study, we collected the clinical histories of 467 patients diagnosed with AML at the University of Maryland Medical Center, including 125 multimodal features recorded between the years 2006–2021. Following rigorous filtration and data interpolation, the curated, unique dynamic dataset comprised 245 patients with 75 clinical predictive features, including patient demographics, genetic and chromosomal abnormalities, disease management strategies, and progression markers (blast percentages), for downstream analyses. Survival curves of different patient cohorts revealed that survival rates were lower among older patients, highest among those with less than 15% blasts, and most favorable for patients receiving stem cell transplant treatments. However, correlation and clustering analyses identified no strong relationships between clinical features and disease progression, as evaluated using both linear and nonlinear methods. To further evaluate predictive performance, we applied supervised ensemble ML multivariable approaches trained on static representations of clinical features. While these methods achieved moderate accuracy in predicting blast percentages, they are limited in modeling the dynamic nature of AML, where disease progression and treatment response evolve over time. These findings from correlation analysis and traditional ML methods highlight the complex interplay between clinical features and AML progression, underscoring the need for advanced computational frameworks capable of learning from temporal clinical data to better capture these complex relationships.

Hence, we sought to identify the mechanisms underlying AML progression by developing a novel predictive methodology to discover dynamic mathematical models from longitudinal clinical data. A high-performance evolutionary computation approach, together with a dynamic mathematical modeling framework, was then developed and applied to the curated clinical dataset, enabling efficient inference of models capable of predicting disease progression. The resulting models, represented as systems of ordinary differential equations (ODEs), included essential gene mutations, regulatory mechanisms, and all numerical parameters, allowing for simulations of the temporal dynamics of AML progression. The discovered models showed minor variations in predicting disease progression while maintaining strong generalization power to accurately capture disease dynamics in novel patients.

Additionally, the discovered models identified key predictive markers and elucidated their mechanistic effects on disease progression, highlighting drivers or mitigators of AML. In particular, the dynamic model presented in Fig. 7 includes many of the factors that leukemia specialists routinely use in clinical interpretation [47]. For example, the presence of cytogenetic abnormalities compared with a normal karyotype is a well-established high-risk feature. Similarly, FLT3-ITD is associated with higher blast counts and represents one of the most common mutations in AML [48, 49]. Many of the features selected in the model are both highly prognostic and prevalent in AML [47]. Therapeutic interventions, such as Ara-C and consolidation regimens, demonstrated a direct negative relationship with blast percentage, while CLAG-M/FLAG-IDA, HMA, and FLT3 inhibitors appeared to activate node *a*, which in turn inhibited blast percentage, reflecting therapeutic benefit. These drugs are proven to be effective and are used as the standard of care in AML management [50]. The combinatoric dynamic insights brought by the quantitative model hold significant potential for improving the diagnosis and treatment of AML in clinical and research settings.

Interestingly, DNMT3A was observed to stimulate node *c*, which increased blast percentage, yet it also inhibited node *b*, a suppressor of node *a*, which itself inhibits blast percentage. This layered regulatory structure may reflect the complex interplay between DNMT3A, a key modulator of DNA methylation [51], and HMA, which reverse DNA methylation changes [51]. Node *a*, thus, may represent an intermediary regulatory hub capturing these opposing effects. These intricate, multi-layered [52] relationships, often nonlinear and context-dependent, are not easily discernible through conventional clinical reasoning or simple correlation analyses, highlighting the unique advantage of computational evolutionary modeling in uncovering hidden biological interactions.

​The proposed methodology was applied to a comprehensive cohort of AML patients, yet it has the potential to be extended to predict treatment outcomes for other acute diseases. Regarding its efficiency, the method begins with random initial models, but as a possible optimization, it could incorporate an initial population of models including pre-established nodes and interactions from known knowledge relevant to disease progression. Incorporating different interpolation techniques could further improve the temporal reconstruction of clinical trajectories, leading to more accurate modeling of disease dynamics. Importantly, evolutionary dynamic modeling may also predict which mutations are likely to emerge, thereby informing the timing of disease monitoring and the early detection of relapse. This could also help identify rational combination therapies aimed at preventing disease recurrence. The predictive power of the model could be further enhanced by incorporating additional molecular features and more sensitive techniques, such as measurable residual disease (MRD) testing. Additionally, the algorithm is currently designed to return a single model per run, although multiple models could provide a more accurate representation of disease progression dynamics. Future work will focus on extending the methodology with evolutionary multi-objective and diversity-preserving algorithms [53] to enable the discovery of a broader set of models—an atlas of regulatory models—capable of predicting AML disease progression.

Further validation of the methodology will be critical for assessing the generalizability of the predictive models. However, curating harmonized longitudinal datasets remains a substantial challenge, particularly in the context of AML. The presented methodology takes temporally structured clinical data across multiple modalities as input, including but not limited to patient demographics, leukemia-specific features, treatment interventions, and a consistent disease progression marker (e.g., blast percentage in this work). Such comprehensive datasets are rarely standardized across institutions or medical centers. Future work will prioritize further validation through collaborative efforts with other institutions or by leveraging emerging harmonized AML datasets as they become available towards predictive comprehensive dynamic models of AML and other diseases progression.​

## Human Ethics

The University of Maryland, Baltimore Institutional Review Board and the University of Maryland, Baltimore County Office of Research Protections and Compliance have certified and approved this research study under the U.S. Department of Health and Human Services regulations for the protection of human subjects in research 45 CFR 46.101(b). This research was conducted under the ethical principles outlined in the April 18, 1979 report of The National Commission for the Protection of Human Subjects of Biomedical and Behavioral Research titled “Ethical Principles and Guidelines for the Protection of Human Subjects of Research,” also known as “The Belmont Report”. Clinical trial number: not applicable. 

## Supplementary Information

Below is the link to the electronic supplementary material.

Supplementary Material 1

## Data Availability

The source code for the inference, simulation, and visualization methods are freely available on GitHub (https://github.com/lobolab/kinetic-leukemia).
