# Supplementary material for: Predicting the Regulatory Dynamics of AML Disease Progression from Longitudinal Multi-Modal Clinical Data
Source: J Med Syst. 2025 Dec 13;49(1):183. doi: 10.1007/s10916-025-02317-6 (PMC12700996; doi:10.1007/s10916-025-02317-6)
Supplement: Supplementary file 1 — Supplementary Material 1 [file 10916_2025_2317_MOESM1_ESM.pdf]

Predicting the Regulatory Dynamics of AML Disease Progression from Longitudinal Multi-Modal Clinical Data

Reza Mousavi, Moaath K. Mustafa Ali, and Daniel Lobo

Supplementary Information

1. Supplementary Materials and Methods 2
   1.1. Data acquisition 2
   1.2. Data filtration 2
   1.3. Data interpolation 2
   1.4. Data analysis 3
   1.5. Model inference algorithm 4
   1.6. Model evaluation 6
   1.7. Implementation 8
   Supplementary references 8
2. Supplementary Figures 9
3. System of Equations 14

1. Supplementary Materials and Methods

1.1. Data acquisition

For this study, an anonymized clinical dataset was curated from the University of Maryland Medical Center, comprising 467 patients diagnosed with acute myeloid leukemia between October 2006 and June 2021. The protocol was approved by the University of Maryland, Baltimore Institutional Review Board and the University of Maryland, Baltimore County Office of Research Protections and Compliance. The patients' ages ranged from 18 to 98 years and comprised a male/female ratio of 1.33. This comprehensive dataset contains detailed, temporally structured clinical information: patient demographics, leukemia-associated genetic and cytogenetic parameters: treatment histories, and a disease progression marker (blast percentages).

1.2. Data filtration

The data curation process involved a rigorous filtration approach to ensure consistency, accuracy, and quality of the collected data for downstream analyses. Patients lacking data on treatments, with inconsistencies in treatment dates, missing genetic mutation data, or fewer than two data points in the disease progression marker (patient blasts) were excluded. In addition, clinical features with missing information were excluded to improve model interpretability and reduce computational complexity. This thorough curation of the clinical dataset ensured robust and accurate analyses. It also improved the efficiency and performance of the evolutionary computation methodology used to infer predictive models.

1.3. Data interpolation

Discrete patient, mutation, and disease progression data points were transformed into dynamic continuous representations using a linear interpolation approach. In this way, linear curves were computed between discrete data points to generate continuous patient trajectories, while preserving the underlying trends in clinical data. Genetic mutations in the collected dataset were recorded as the percentage of allele frequency (AF), defined as the proportion of sequencing reads containing a specific mutation relative to the total reads at the corresponding genomic location. These percentages provide a quantitative measure of mutation burden within each sample. In the collected data, two types of blast measurements were available: PB blasts and BM blasts. Due to the limited availability of these values across time, we applied a strategy to combine them.

Specifically, when both PB and BM blast values were clinically recorded at the same time point, their average was calculated and used as the representative blast value. This averaging smoothed transitions between time points and improved continuity in blast percentage trajectories for modeling. If only one type was available, it was directly used as the blast percentage for that time point. This approach facilitated the creation of smoother, more complete blast trajectories, minimizing the impact of missing data and enhancing the accuracy of longitudinal analyses of AML progression. Cytogenetic abnormality data were categorized and transformed into binary continuous temporal variables. Significant cytogenetic labels (e.g., abnormal metaphase findings, FISH abnormalities, persistent abnormalities, and partial remission) were assigned a numeric value between 0 and 100. Treatment intervention data points were transformed into binary (a particular treatment being applied or not) continuous temporal functions. Such temporal binary data differentiated between the treated and untreated periods, allowing the model to accurately assess the impact of each intervention on AML progression. By integrating and normalizing these diverse temporal data, we constructed a longitudinal continuous multimodal dataset suitable for inferring mechanistic dynamic models to predict AML disease progression.

1.4. Data analysis

After filtering, the curated multimodal AML dataset was explored to assess patterns and dynamics across clinical variables, treatment strategies, genetic mutations, and the disease progression marker (blast percentage). Venn diagrams were constructed by transforming mutation and treatment strategies into binary data (occurrence or absence during the clinical history of a patient) and visualizing their overlap among all patients. Cumulative survival plots were generated using the Kaplan-Meier method [1], grouping patients by age, genetic mutations, treatment interventions, and the disease progression marker (blast percentage). For the age feature, survival analysis was based on the age at diagnosis, while mutation and treatment strategies were assessed as binary data (occurrence or absence during the clinical history of a patient). The disease progression marker was analyzed using continuous values calculated as the area under the curve (AUC) normalized by the clinical history duration for the patient. Statistical significance between survival groups was computed using a log-rank test.

To investigate potential correlations between patient features and the disease progression marker (blast percentage), dimensionality reduction and clustering techniques were applied. Principal

Component Analysis (PCA) and Uniform Manifold Approximation and Projection (UMAP) were applied to explore correlations between the clinical features and the progression marker. Additionally, a clustered heatmap was generated to provide a detailed overview of feature clustering and its potential association with disease progression. To complement these exploratory analyses, we applied supervised ensemble learning methods—Random Forest and Gradient Boosting [2]—to predict blast percentages using all clinical features listed in Table 1. All analyses, including PCA, UMAP, heatmap clustering, and ensemble modeling, were performed on static data, using continuous values derived by calculating AUC of features normalized by the clinical history duration.

1.5. Model inference algorithm

A *de novo* inference algorithm was developed using high-performance evolutionary computation to efficiently discover dynamic models. These models include the number of nodes, their regulatory interactions, and parameters derived from the clinical dataset. The method can infer the topology and parameters of a dynamic predictive model defined by a system of ordinary differential equations (ODEs), which can accurately simulate AML disease progression, defined as the blast percentage over time. Taking the curated longitudinal multimodal dataset as input, the method can provide a reliable and interpretable framework for understanding and predicting individual disease trajectories.

The automatic approach leverages evolutionary computation to infer a model that recapitulates the disease progression dynamics. The algorithm iteratively evolves a population of candidate predictive models through reproduction, fitness assessment, and selection, ultimately returning an optimal model that best represents the underlying dynamics. An island distribution strategy was employed to enhance parallel processing, while promoting robustness and diversity among candidate models. New models are generated through stochastic combinations of existing models in addition to random mutations in their parameters, interactions, and nodes. A crossover operator creates two child models by randomly merging models from two parents within the population, redistributing nodes and regulatory interactions without duplicating nodes or altering kinetic parameters. Subsequently, a mutation operator may add or remove nodes and regulatory interactions, or modify parameters within predefined ranges sampled from a uniform random distribution. The output node (blast percentage) is always preserved. The algorithm is biased

towards simpler models by assigning a higher probability of deletion mutations compared to duplication mutations, thereby preventing model bloating [3].

The selection mechanism is based on deterministic crowding [4], replacing new offspring only if they have equal or improved fitness compared with their closest parent models. The algorithm iterates until it finds a model with zero error, after which it continues until 2000 additional generations are completed without any reduction in the model complexity (number of edges plus number of nodes). The evolutionary computation method was run with the following meta-parameters: crossover rate, 75%; mutation rate, 1%; link/gene duplication rate, 1%; link/gene deletion rate, 1.5%. Each run used 32 subpopulations (islands), with each consisting of 64 individuals. To enhance diversity and exploration in the search space, islands were randomly paired, and their models were swapped every 250 generations.

A disease progression model simulator was developed to predict patient longitudinal dynamics using a system of ODEs. Models comprise nodes representing the dynamic levels of the patient, mutation, treatment, and disease progression data, together with intermediate nodes integrating these complex signals. Hence, nodes in a model are categorized into three groups: (1) input nodes representing patient information, genetic mutations and abnormalities, and treatment interventions that follow the dynamic continuous values in a patient as curated in the clinical dataset; (2) intermediate nodes representing integration steps of signal dynamics; and (3) the disease progression output node predicted by the model as the blast percentage over time. A model also includes all the regulatory interactions between the nodes and defines the type and parameters of each interaction. Input nodes can regulate other nodes but cannot be regulated by either themselves or other nodes, as they follow the dynamics interpolated from the patient's clinical data. In contrast, intermediate nodes and the output node can be regulated by other nodes and themselves, as they change their values according to the equations defined in the model and evolve with the inference methodology.

Each intermediate node and output node in the predictive model includes two parameters: production and decay rates. Regulatory interactions are characterized as either positive or negative and modeled using a Hill function with two parameters: the Hill coefficient and half-response values. Nodes can receive regulations from multiple nodes simultaneously, and these regulatory

inputs can be classified as either necessary or sufficient. Necessary positive regulations are combined using a multiplication operator, whereas sufficient positive regulations use both multiplication and summation. Negative regulations are combined exclusively using multiplication operators. This approach provides a robust and interpretable mechanism for predicting AML progression dynamics by incorporating patient-specific factors and dynamic regulatory interactions.

Thus, the level n_i of an intermediate or output node i is given by

$$\frac{dn_i}{dt} = \rho_i R_i - \lambda_i n_i$$

where ρ_i is the production constant and λ_i is the decay constant. The regulatory term R_i is modeled using a Hill function for each regulatory interaction that affects the node n_i. For example, the following equation describes a regulatory term R affecting the node n_i when it is regulated by two sufficient positive nodes r and g, two necessary positive nodes p and y, and a negative node o:

$$\mathrm{R} = \frac{\beta + [\left(\frac{r}{\alpha_1}\right)^{\eta_1}\left(\frac{g}{\alpha_2}\right)^{\eta_2} + \left(\frac{r}{\alpha_1}\right)^{\eta_1} + \left(\frac{g}{\alpha_2}\right)^{\eta_2}]\left(\frac{y}{\alpha_3}\right)^{\eta_3}\left(\frac{p}{\alpha_4}\right)^{\eta_4}}{\left[1+\left(\frac{r}{\alpha_1}\right)^{\eta_1}\right]\left[1+\left(\frac{g}{\alpha_2}\right)^{\eta_2}\right]\left[1+\left(\frac{y}{\alpha_3}\right)^{\eta_3}\right]\left[1+\left(\frac{p}{\alpha_4}\right)^{\eta_4}\right]\left[1+\left(\frac{o}{\alpha_5}\right)^{\eta_5}\right]}$$

where η_i are the Hill coefficients, and α_i are the half-response values. Model parameter ranges were as follows: Hill coefficient (1,10), half-response value (0.01,100), decay constant (0.1, 1), and production constant (0, 100).

1.6. Model evaluation

The fitness of a model measures its capability to accurately predict disease progression dynamics (blast percentage) when simulated using the clinical data of each patient in the training set. This accuracy is quantified through an error function. The error for a candidate model is calculated by simulating the disease dynamics for each patient and computing the average Euclidean distances between the actual and predicted blast percentage values across their entire clinical history. To minimize overfitting and increase the discovery of simpler models, two parameters, represented by the local threshold α and global threshold β, are included in the error function. These thresholds ensure that models with concentration scores below these limits are assigned an error of zero. This strategy avoids adding unnecessary complexity to models, so that extra intermediate nodes or regulatory interactions are included only if they significantly improve predictive performance. In

this way, the error of a model with respect to an input dataset comprising the curated clinical data of a set of patients is calculated as

$$\text{error} = \left(\frac{1}{p} \sum_{i=1}^{p} \sqrt{\frac{1}{n} \sum_{j=1}^{n} \left(\left(|y_{i,j} - \hat{y}_{i,j}| - \beta \right)^{+} \right)^{2}} - \alpha \right)^{+}$$

where p and n are the number of patients and the number of data points for the blast marker in patient i, respectively, $y_{i,j}$ and $\hat{y}_{i,j}$ are the recorded patient and model-predicted blast values at time point j for patient i, α and β are the local and global thresholds, respectively. For all the runs, the α and β threshold parameters were set to 10 and 1, respectively. The function $(x)^{+}$ represents the positive part function, which outputs 0 if x is negative and x if x is nonnegative.

To evaluate the model performance on the testing set, the observed patient blast values were compared with the model's predicted values using the root-mean-square error (RMSE), normalized root-mean-square error ($RMSE_n$), coefficient of determination (R^2), mean absolute percentage error (MAPE), and mean absolute error (MAE), which were computed as

$$RMSE = \sqrt{\frac{\sum_{i=1}^{n} (y_i - \hat{y}_i)^2}{n}}$$

$$RMSE_n = \frac{RMSE \times 100}{\bar{y}}$$

$$R^2 = \frac{\sum_{i=1}^{n} (y_i - \bar{y})(\hat{y}_i - \bar{\hat{y}})}{\sqrt{\sum_{i=1}^{n} (y_i - \bar{y})^2 \sum_{i=1}^{n} (\hat{y}_i - \bar{\hat{y}})^2}}$$

$$MAPE = \frac{1}{n} \sum_{i=1}^{n} \left| \frac{y_i - \hat{y}_i}{y_i} \right| \times 100$$

$$MAE = \frac{1}{n} \sum_{i=1}^{n} |y_i - \hat{y}_i|$$

where n is the number of data points, $\mathrm{y_i}$ and $\mathrm{\hat{y}_i}$ denote the observed and model-predicted values at the time point i, respectively, and $\bar{\mathrm{y}}$ and $\bar{\hat{\mathrm{y}}}$ represent the means of observed and predicted values, respectively. The R^2 was calculated using linear regression between the predicted and the actual blast percentages. Higher R^2 and lower RMSE, $RMSE_n$, MAPE, and MAE values indicate a closer

fit between the predicted and actual blast values, reflecting a higher accuracy of the model's predictions.

1.7. Implementation

Data analysis and visualization were conducted using a diverse set of Python libraries. Survival analysis was conducted using the *lifelines* library, while hierarchical clustering was carried out using the *scipy* library. The *venn* library was used to generate the Venn diagrams. Dimensionality reduction techniques including Principal Component Analysis (PCA) and Uniform Manifold Approximation and Projection (UMAP), and supervised ensemble learning methods including Random Forest Regressor and Gradient Boosting Regressor, were implemented with the *scikit-learn* library. All visualizations were created with the *seaborn* and *matplotlib* libraries, providing clear and effective graphical representations of the results. We implemented the inference and model simulation methodology in C + + with the standard Eigen (Gaël Guennebaud, Benoît Jacob, and others), Qt (The Qt Company Ltd.), and Qwt (Uwe Rathmann and Josef Wilgen) libraries. A generalized Runge-Kutta eighth-order solver with an adaptive step size [5] was developed to numerically solve the system of ODEs. The implementation leveraged 64 parallel threads and was executed on a server equipped with two 32-core Intel Xeon Gold 6548Y+ CPUs and 512GB of RAM to assess its performance.

Supplementary references

1. Bland JM, Altman DG (1998) Survival probabilities (the Kaplan-Meier method). BMJ 317:1572–1580. https://doi.org/10.1136/bmj.317.7172.1572
2. Mousavi R, Eftekhari M (2015) A new ensemble learning methodology based on hybridization of classifier ensemble selection approaches. Applied Soft Computing 37:652–666. https://doi.org/10.1016/j.asoc.2015.09.009
3. Luke S, Panait L (2006) A Comparison of Bloat Control Methods for Genetic Programming. Evolutionary Computation 14:309–344. https://doi.org/10.1162/evco.2006.14.3.309
4. Mahfoud SW (1992) Crowding and preselection revisited. In: Manner R, Manderick B (eds) Parallel Problem Solving from Nature 2. Elsevier, pp 27–36
5. Press WH (2007) Numerical Recipes 3rd Edition: The Art of Scientific Computing. Cambridge University Press

2. Supplementary Figures

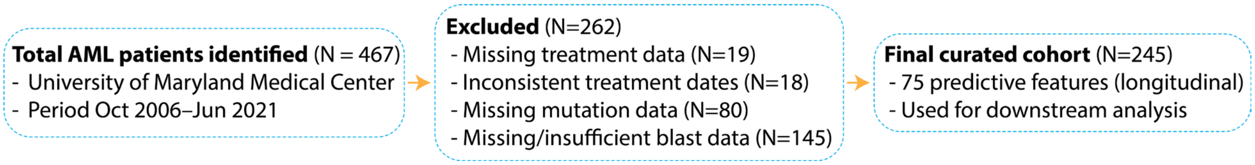

Supplementary Figure 1. Patient selection and filtration flowchart. Data from 467 AML patients were collected from the University of Maryland Medical Center. A total of 262 patients were excluded due to missing treatment data, inconsistencies in treatment dates, missing genetic mutation data, or insufficient disease progression data (blast percentages). The final analytic cohort included 245 patients with complete and high-quality multimodal clinical data used for downstream predictive modeling.

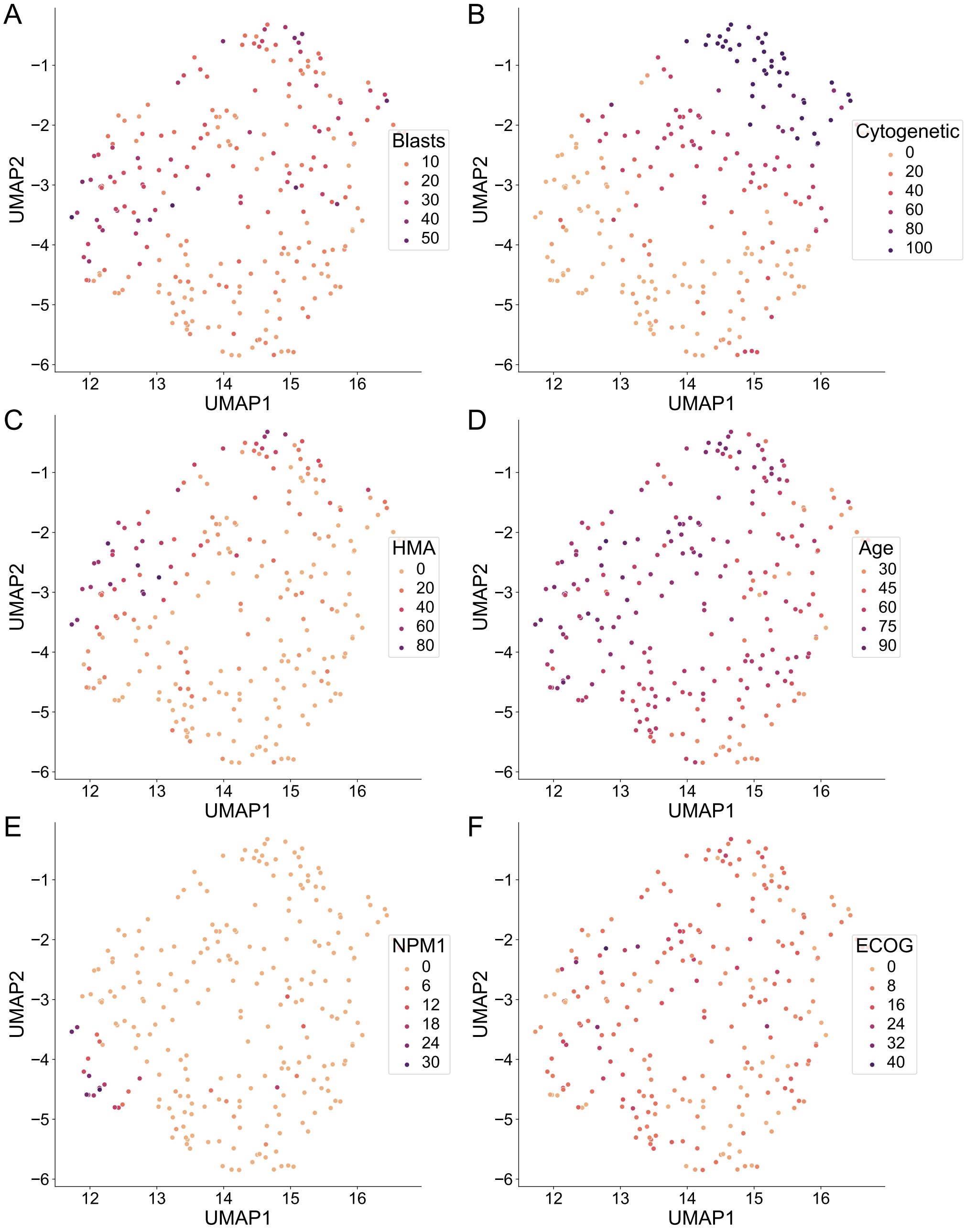

Supplementary Figure 2. UMAP plots highlight the relationships between clinical, molecular, and disease progression features in the curated AML patient dataset. A. Blast percentage, representing the marker of disease progression. **B.** Cytogenetic abnormalities, a molecular feature reflecting changes in chromosome number or structure. **C.** Hypomethylating Agents (HMA), a class of drugs used in AML treatment. **D.** Age, measured at the time of diagnosis. **E.** Mutations in *NPM1*, as commonly observed in AML, **F.** ECOG performance status, a clinical feature representing a functional measure of daily activity and self-care ability. For each feature, except age, data were derived from dynamic variables and calculated as the area under the curve (AUC) divided by the clinical history duration. Each point represents an individual patient, plotted according to their distribution across the first two UMAP dimensions. Points are colored by the respective clinical or disease feature to highlight potential correlations.

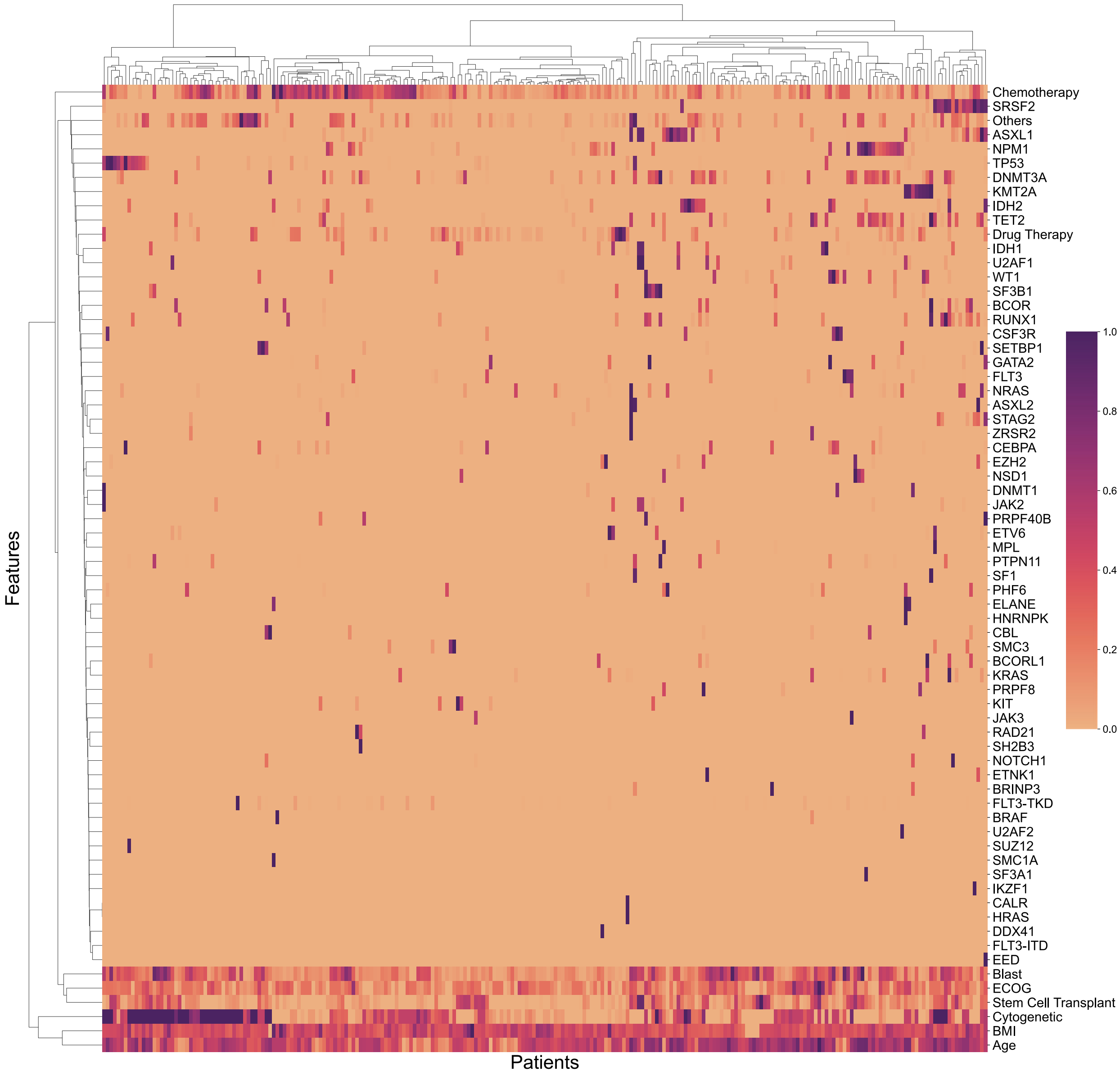

Supplementary Figure 3. Clustered heatmap of patients based on all clinical, molecular, and disease progression features. Clinical features include age, BMI, and ECOG performance status; molecular features include cytogenetic abnormalities and mutation status of genes such as FLT3, NPM1, IDH1, and TP53; treatment features include chemotherapy, targeted drug therapy, stem cell transplantation, and other interventions. For each feature, data were derived from continuous variables, calculated as the area under the curve (AUC) divided by the clinical history duration. The color indicates normalized values across the cohort for each feature, with dark and light colors representing high and low values, respectively.

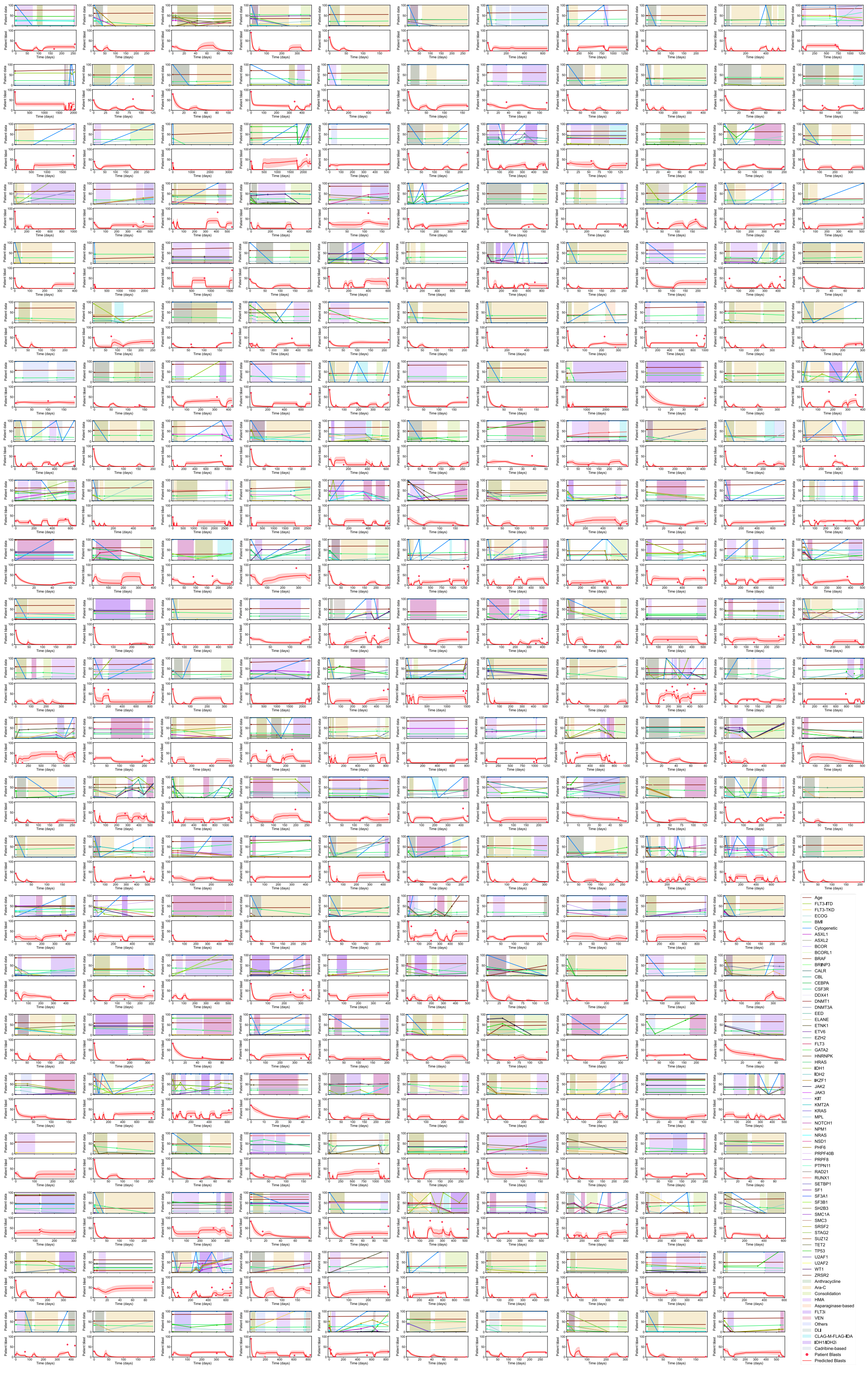
Patient data
Patient blast
Time (days)
Age
FLT3-ITD
FLT3-TKD
ECOG
BMI
Cytogenetic
ASXL1
ASXL2
BCOR
BCORL1
BRAF
BRINP3
CALR
CBL
CEBPA
CSF3R
DDX41
DNMT1
DNMT3A
EED
ELANE
ETNK1
ETV6
EZH2
FLT3
GATA2
HNRNPK
HRAS
IDH1
IDH2
IKZF1
JAK2
JAK3
KIT
KMT2A
KRAS
MPL
NOTCH1
NPM1
NRAS
NSD1
PHF6
PRPF40B
PRPF8
PTPN11
RAD21
RUNX1
SETBP1
SF1
SF3A1
SF3B1
SH2B3
SMC1A
SMC3
SRSF2
STAG2
SUZ12
TET2
TP53
U2AF1
U2AF2
WT1
ZRSR2
Anthracycline
Ara-C
Consolidation
HMA
Asparaginase-based
FLT3i
VEN
Others
DLI
CLAG-M-FLAG-IDA
IDH1/IDH2i
Cadribine-based
Patient Blasts
Predicted Blasts

Supplementary Figure 4. Predicted disease progression dynamics for all patients in the curated AML dataset. The top panel for each patient displays input features categorized into three modalities: patient information, leukemia parameters, and disease management interventions, as detailed in Table 1 in the main text. The bottom panel for each patient illustrates the predicted longitudinal dynamics of disease progression. The line and shaded region represent the average and standard deviation, respectively, of error values across all models.

3. System of Equations

System of equations governing the model illustrated in Figure 7a:

$$\frac{dblasts}{dt} = 4.8 \cdot \frac{\left(\left(\left(\left(99 \cdot Age^{3.2} \cdot 1.6 \cdot FLT3-ITD^{9.1} + 99 \cdot Age^{3.2} + 1.6 \cdot FLT3-ITD^{9.1}\right) \cdot 97 \cdot Cytogenetic^{1.2} + \left(99 \cdot Age^{3.2} \cdot 1.6 \cdot FLT3-ITD^{9.1} + 99 \cdot Age^{3.2} + 1.6 \cdot FLT3-ITD^{9.1}\right) + 97 \cdot Cytogenetic^{1.2}\right) \cdot 2.8 \cdot MPL^{6.4} + \left(\left(99 \cdot Age^{3.2} \cdot 1.6 \cdot FLT3-ITD^{9.1} + 99 \cdot Age^{3.2} + 1.6 \cdot FLT3-ITD^{9.1}\right) \cdot 97 \cdot Cytogenetic^{1.2} + \left(99 \cdot Age^{3.2} \cdot 1.6 \cdot FLT3-ITD^{9.1} + 99 \cdot Age^{3.2} + 1.6 \cdot FLT3-ITD^{9.1}\right) + 97 \cdot Cytogenetic^{1.2}\right) + 2.8 \cdot MPL^{6.4}\right) \cdot 0.63 \cdot SETBP1^{6.6} + \left(\left(\left(99 \cdot Age^{3.2} \cdot 1.6 \cdot FLT3-ITD^{9.1} + 99 \cdot Age^{3.2} + 1.6 \cdot FLT3-ITD^{9.1}\right) \cdot 97 \cdot Cytogenetic^{1.2} + \left(99 \cdot Age^{3.2} \cdot 1.6 \cdot FLT3-ITD^{9.1} + 99 \cdot Age^{3.2} + 1.6 \cdot FLT3-ITD^{9.1}\right) + 97 \cdot Cytogenetic^{1.2}\right) \cdot 2.8 \cdot MPL^{6.4} + \left(\left(99 \cdot Age^{3.2} \cdot 1.6 \cdot FLT3-ITD^{9.1} + 99 \cdot Age^{3.2} + 1.6 \cdot FLT3-ITD^{9.1}\right) \cdot 97 \cdot Cytogenetic^{1.2} + \left(99 \cdot Age^{3.2} \cdot 1.6 \cdot FLT3-ITD^{9.1} + 99 \cdot Age^{3.2} + 1.6 \cdot FLT3-ITD^{9.1}\right) + 97 \cdot Cytogenetic^{1.2}\right) + 2.8 \cdot MPL^{6.4}\right) + 0.63 \cdot SETBP1^{6.6}\right) \cdot 4 \cdot c^{9.7} + \left(\left(\left(\left(99 \cdot Age^{3.2} \cdot 1.6 \cdot FLT3-ITD^{9.1} + 99 \cdot Age^{3.2} + 1.6 \cdot FLT3-ITD^{9.1}\right) \cdot 97 \cdot Cytogenetic^{1.2} + \left(99 \cdot Age^{3.2} \cdot 1.6 \cdot FLT3-ITD^{9.1} + 99 \cdot Age^{3.2} + 1.6 \cdot FLT3-ITD^{9.1}\right) + 97 \cdot Cytogenetic^{1.2}\right) \cdot 2.8 \cdot MPL^{6.4} + \left(\left(99 \cdot Age^{3.2} \cdot 1.6 \cdot FLT3-ITD^{9.1} + 99 \cdot Age^{3.2} + 1.6 \cdot FLT3-ITD^{9.1}\right) \cdot 97 \cdot Cytogenetic^{1.2} + \left(99 \cdot Age^{3.2} \cdot 1.6 \cdot FLT3-ITD^{9.1} + 99 \cdot Age^{3.2} + 1.6 \cdot FLT3-ITD^{9.1}\right) + 97 \cdot Cytogenetic^{1.2}\right) + 2.8 \cdot MPL^{6.4}\right) \cdot 0.63 \cdot SETBP1^{6.6} + \left(\left(\left(99 \cdot Age^{3.2} \cdot 1.6 \cdot FLT3-ITD^{9.1} + 99 \cdot Age^{3.2} + 1.6 \cdot FLT3-ITD^{9.1}\right) \cdot 97 \cdot Cytogenetic^{1.2} + \left(99 \cdot Age^{3.2} \cdot 1.6 \cdot FLT3-ITD^{9.1} + 99 \cdot Age^{3.2} + 1.6 \cdot FLT3-ITD^{9.1}\right) + 97 \cdot Cytogenetic^{1.2}\right) \cdot 2.8 \cdot MPL^{6.4} + \left(\left(99 \cdot Age^{3.2} \cdot 1.6 \cdot FLT3-ITD^{9.1} + 99 \cdot Age^{3.2} + 1.6 \cdot FLT3-ITD^{9.1}\right) \cdot 97 \cdot Cytogenetic^{1.2} + \left(99 \cdot Age^{3.2} \cdot 1.6 \cdot FLT3-ITD^{9.1} + 99 \cdot Age^{3.2} + 1.6 \cdot FLT3-ITD^{9.1}\right) + 97 \cdot Cytogenetic^{1.2}\right) + 2.8 \cdot MPL^{6.4}\right) + 0.63 \cdot SETBP1^{6.6}\right) + 4 \cdot c^{9.7}}{\left(1 + 99 \cdot Age^{3.2}\right) \cdot \left(1 + 1.6 \cdot FLT3-ITD^{9.1}\right) \cdot \left(1 + 97 \cdot Cytogenetic^{1.2}\right) \cdot \left(1 + 2.8 \cdot MPL^{6.4}\right) \cdot \left(1 + 0.63 \cdot SETBP1^{6.6}\right) \cdot \left(1 + 61 \cdot a^{1.6}\right) \cdot \left(1 + 4 \cdot c^{9.7}\right) \cdot \left(1 + 72 \cdot Ara-C^{8.7}\right) \cdot \left(1 + 31 \cdot Consolidation^{2.1}\right) \cdot \left(1 + 73 \cdot \frac{IDH1}{IDH2i}^{8.8}\right)} - 0.13 \cdot blasts$$

$$\frac{da}{dt} = 43 \cdot \frac{\left(82 \cdot HMA^{8.6} \cdot 75 \cdot FLT3i^{5} + 82 \cdot HMA^{8.6} + 75 \cdot FLT3i^{5}\right) \cdot 73 \cdot CLAG-M-FLAG-IDA^{2.5} + \left(82 \cdot HMA^{8.6} \cdot 75 \cdot FLT3i^{5} + 82 \cdot HMA^{8.6} + 75 \cdot FLT3i^{5}\right) + 73 \cdot CLAG-M-FLAG-IDA^{2.5}}{\left(1 + 18 \cdot KIT^{7.7}\right) \cdot \left(1 + 82 \cdot HMA^{8.6}\right) \cdot \left(1 + 75 \cdot FLT3i^{5}\right) \cdot \left(1 + 49 \cdot Others^{7.5}\right) \cdot \left(1 + 73 \cdot CLAG-M-FLAG-IDA^{2.5}\right) \cdot \left(1 + 43 \cdot b^{9.9}\right)} - 0.23 \cdot a$$

$$\frac{db}{dt} = 57 \cdot \frac{\left(28 \cdot FLT3^{7.9} \cdot 36 \cdot NSD1^{8.3} + 28 \cdot FLT3^{7.9} + 36 \cdot NSD1^{8.3}\right) \cdot 12 \cdot PTPN11^{2.8} + \left(28 \cdot FLT3^{7.9} \cdot 36 \cdot NSD1^{8.3} + 28 \cdot FLT3^{7.9} + 36 \cdot NSD1^{8.3}\right) + 12 \cdot PTPN11^{2.8}}{\left(1 + 28 \cdot FLT3^{7.9}\right) \cdot \left(1 + 36 \cdot NSD1^{8.3}\right) \cdot \left(1 + 12 \cdot PTPN11^{2.8}\right) \cdot \left(1 + 81 \cdot DNMT3A^{9.2}\right)} - 0.38 \cdot b$$

$$\frac{dc}{dt} = 13 \cdot \frac{\left(70 \cdot DNMT3A^{4.5} \cdot 21 \cdot NPM1^{6.3} + 70 \cdot DNMT3A^{4.5} + 21 \cdot NPM1^{6.3}\right) \cdot 9 \cdot PRPF40B^{7.5} + \left(70 \cdot DNMT3A^{4.5} \cdot 21 \cdot NPM1^{6.3} + 70 \cdot DNMT3A^{4.5} + 21 \cdot NPM1^{6.3}\right) + 9 \cdot PRPF40B^{7.5}}{\left(1 + 70 \cdot DNMT3A^{4.5}\right) \cdot \left(1 + 21 \cdot NPM1^{6.3}\right) \cdot \left(1 + 9 \cdot PRPF40B^{7.5}\right) \cdot \left(1 + 17 \cdot PTPN11^{4}\right)} - 0.11 \cdot c$$
